# Biomimetic Polymerization of Tellurocysteine: Breaking the Natural Amino Acid Radioprotection Limitation

**DOI:** 10.1002/advs.202600010

**Published:** 2026-04-07

**Authors:** Wei Chen, Hanjie Zhu, Yue Zhang, Yuqing Qiao, Ruotong Deng, Huaping Xu, Wei Cao

**Affiliations:** ^1^ College of Chemistry Key Laboratory of Radiopharmaceuticals of the Ministry of Education Beijing Normal University Beijing China; ^2^ Key Lab of Organic Optoelectronics & Molecular Engineering Department of Chemistry Tsinghua University Beijing China; ^3^ School of Physics and Astronomy Beijing Normal University Beijing China; ^4^ Institute of Catalysis for Energy and Environment College of Chemistry & Chemical Engineering Shenyang Normal University Shenyang China

**Keywords:** amino acids, melanin, polymers, radioprotection, tellurium

## Abstract

Radioprotection remains a critical challenge in biomedicine and space exploration. As the fundamental building blocks of organisms, amino acids are gaining momentum in chemical design for in vivo radioprotection, yet their low atomic number (*Z*) and rapid metabolism restrict practical applications. This study addresses these limitations through the melanin‐inspired polymerization of the higher *Z*‐tellurocysteine. Motivated by the superior catalytic activity and higher *Z* of tellurium over selenium in both enzyme mimics and microbial systems, we hypothesized that tellurium‐containing amino acid polymers could demonstrate enhanced photon interaction and radical scavenging. The exceptional nucleophilic substitution capability of tellurocysteine, which arises from its soft polarizable character, drives its bisubstitution with *o*‐benzoquinone. The heteroatom enrichment and high‐*Z* effect make the novel materials far exceed natural amino acid polymers in radiation shielding. The melanin‐mimetic polymeric structure demonstrates enhanced radiation stability and broad‐spectrum free radical scavenging ability. Following oral administration, the tellurocysteine‐based polymers achieve prolonged intestinal retention, mitigating radiation‐induced intestinal injury. Our work establishes a new paradigm in amino acid engineering, demonstrating how strategic non‐metallic heavy atom incorporation can transform biological molecules into advanced radioprotective materials. This approach opens possibilities for developing next‐generation, amino acid‐derived agents with tailored pharmacokinetics and multifunctional activity.

## Graphical abstract

Inspired by natural amino acid polymer‐melanin systems, this study strategically incorporated tellurocysteine to develop advanced radiation‐shielding materials. This approach transformed the primary interaction mechanism between melanin‐based materials and incident photons from Compton scattering to the photoelectric effect. Tellurocysteine‐polymerized melanin‐like nanoparticles (TeMNPs) significantly attenuated ionizing radiation energy, maintained structural integrity under high‐dose radiation exposure, and effectively scavenged various free radicals. Biological experiments revealed the excellent radioprotection effect of TeMNPs at the cellular level and animal level.

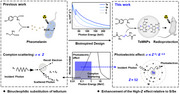

## 1 Introduction

The rapid advancement of the nuclear industry, nuclear medicine, and space travel has significantly increased the risk of radiation exposure [1, 2, 3]. NASA's twins study has revealed the permanent DNA mutations caused by space radiation, suggesting that humans are not yet prepared for space missions that may span years or even decades [3]. Although amifostine is approved by the FDA as a radioprotective agent, its limited administration routes and its side effects (e.g., hypotension, fever, and vomiting) largely restrict its applicability in mitigating radiation‐induced damage [4, 5]. Therefore, there is an urgent need for the chemical design of radioprotective agents that are both safe and effective.

Recent studies have revealed that amino acids and their derivatives play a crucial role in radioprotection [6, 7, 8, 9, 10]. The tryptophan metabolites have been confirmed to provide long‐term radioprotection in vivo [6]. γ‐Amino butyric acid has been shown to inhibit radiation‐induced mitochondrial oxidative stress and subsequent apoptosis [11]. However, small‐molecule amino acids face skepticism due to their rapid clearance, instability, and poor radiation resistance. Incorporating small‐molecule drugs into polymeric structures presents a potential solution to these limitations [12, 13, 14]. For instance, polycysteine has been shown to enhance its DNA‐binding capacity, thereby mitigating radiation‐induced DNA damage and demonstrating radioprotective efficacy [10]. In fact, to survive in environments with high levels of radiation, microorganisms have developed substantial amounts of amino acid‐derived polymers (melanin) [9, 15]. Melanin plays a crucial protective role against radiation by scavenging free radicals and trapping Compton recoil electrons [16, 17]. As the *Z* of a material increases, the dominant interaction mechanism within the ∼1.5 MeV photon energy range shifts toward the photoelectric effect from Compton scattering. Because the Compton scattering cross‐section is linearly proportional to *Z*, while the photoelectric effect cross‐section scales with *Z* ^4^^−^^5^, we hypothesize that increasing the *Z* of melanin will boost the attenuation cross‐section. However, for natural melanin, the critical limitation in the interaction with photons arises from the inherent light‐element composition.

While the natural selenoprotein glutathione peroxidase (GPx) is essential for cellular antioxidant defense, tellurium‐containing amino acid‐based GPx mimics (Telluroprotein) appear to demonstrate significantly enhanced catalytic activity over their selenium counterparts [18, 19, 20, 21]. Due to the valence electron structure of tellurium, which endows it with a variety of oxidation states, resulting in reversible redox properties, tellurium‐based polymers have emerged as a highly promising antioxidant platform with broad application prospects. Moreover, positioned below selenium in Group 16 (chalcogens), tellurium has a greater atomic number (Te: 52 vs. Se: 34) [22, 23]. Therefore, we propose that chemically‐synthesized tellurium‐containing amino acid‐based polymers may demonstrate superior radioprotective properties. Critically, realizing these advantages requires overcoming tellurium's paradoxical chemistry. Incorporating Te into organic frameworks requires harsh conditions (e.g., alkyl lithium reagents) [23, 24], often incompatible with biomaterial late‐stage functionalization. Efficient chemical methodologies for polymerizing unstable tellurocysteine remain lacking. Plus, the stability of the relatively weak bonds involving tellurium under high‐dose irradiation is a concern [24, 25].

**SCHEME 1:**
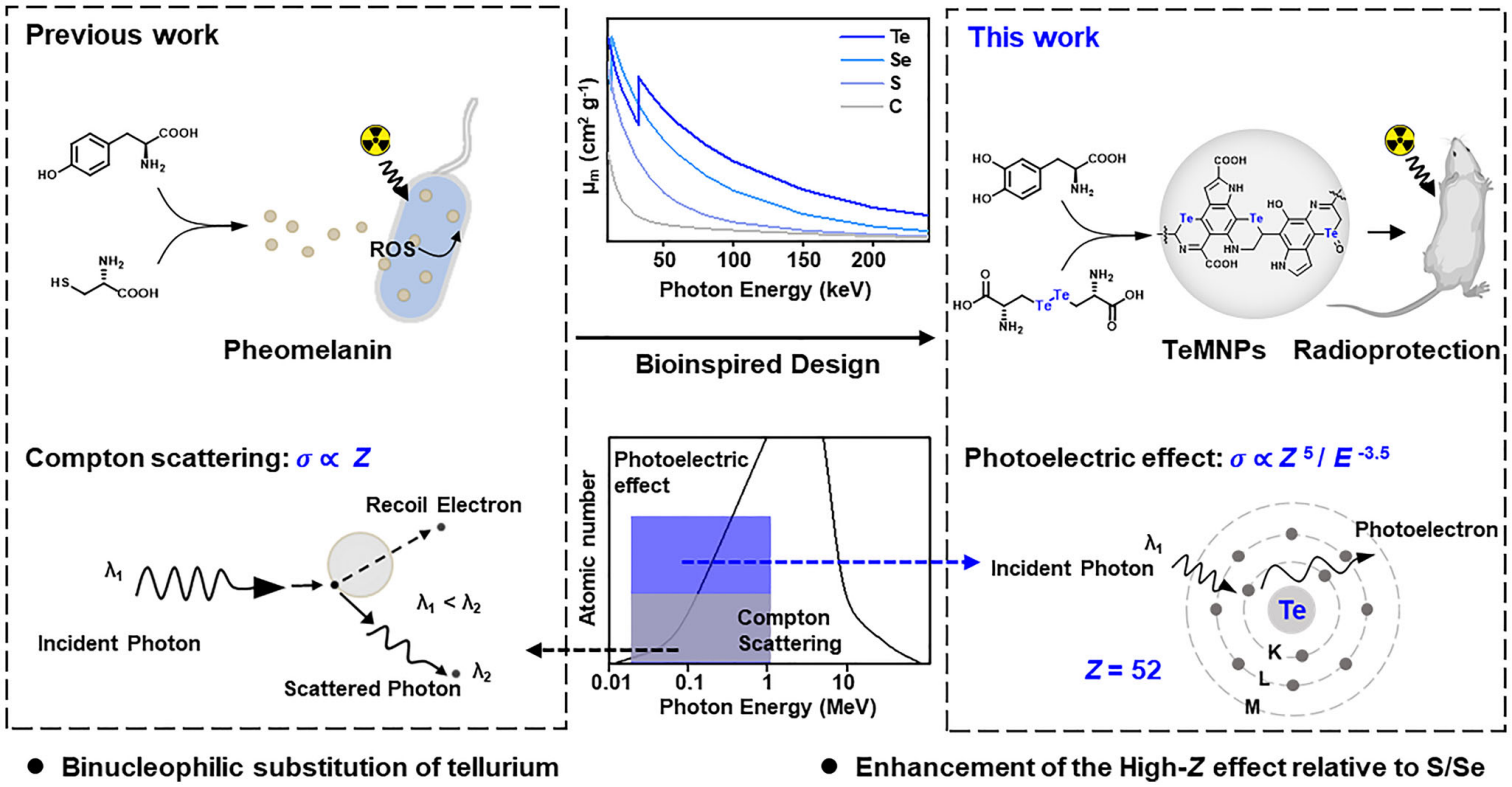
Illustration of the bio‐inspired synthesis of TeMNPs and the proposed mechanisms against radiation. Due to the increased average atomic number of melanin, the primary interaction between radiation photons and melanin shifts from Compton scattering to the photoelectric effect. The photoelectric effect cross‐section is approximately proportional to *Z* ^4^^−^^5^, whereas the Compton scattering cross‐section exhibits only a linear dependence on atomic number. Consequently, the novel melanin synthesized through tellurocysteine polymerization demonstrates exceptional radiation protection performance.

Herein, we report the polymerization of tellurocysteine under facile, organic‐solvent‐free conditions. This is the first tellurium‐containing melanin reported to date. Tellurium exhibits strong nucleophilic substitution reactivity, enabling tellurocysteine to undergo bisubstitution with *o‐*benzoquinone during oxidative polymerization in contrast to its sulfur and selenium counterparts [9, 26]. This helps increase the equivalent average atomic number of the tellurocysteine‐polymerized melanin‐like nanoparticles (TeMNPs). Compared to all known natural amino acids, tellurocystine possesses the highest average atomic number, endowing TeMNPs with unparalleled capabilities in attenuating radiation compared to natural melanin. TeMNPs maintained structural stability even under high‐dose irradiation. Tellurocysteine possesses a low redox potential, enabling tellurocysteine residue‐containing polymers to participate in unique biologically based redox reactions, as has been reported in microbial systems [18, 21]. TeMNPs exhibited exceptional antioxidant properties and were efficiently internalized by cells to mitigate radiation‐induced oxidative stress and DNA damage. Furthermore, TeMNPs were orally administrable, promoting intestinal epithelial regeneration, maintaining gut barrier integrity, and suppressing tissue inflammation to alleviate radiation‐induced injury in mice (Scheme 1). This study provides a unique strategy for advancing amino acid‐based derivatives and opens new avenues for next‐generation radioprotective agents.

## 2 Results and Discussion

### 2.1 Chemical Synthesis and Characterization of TeMNPs

**FIGURE 1:**
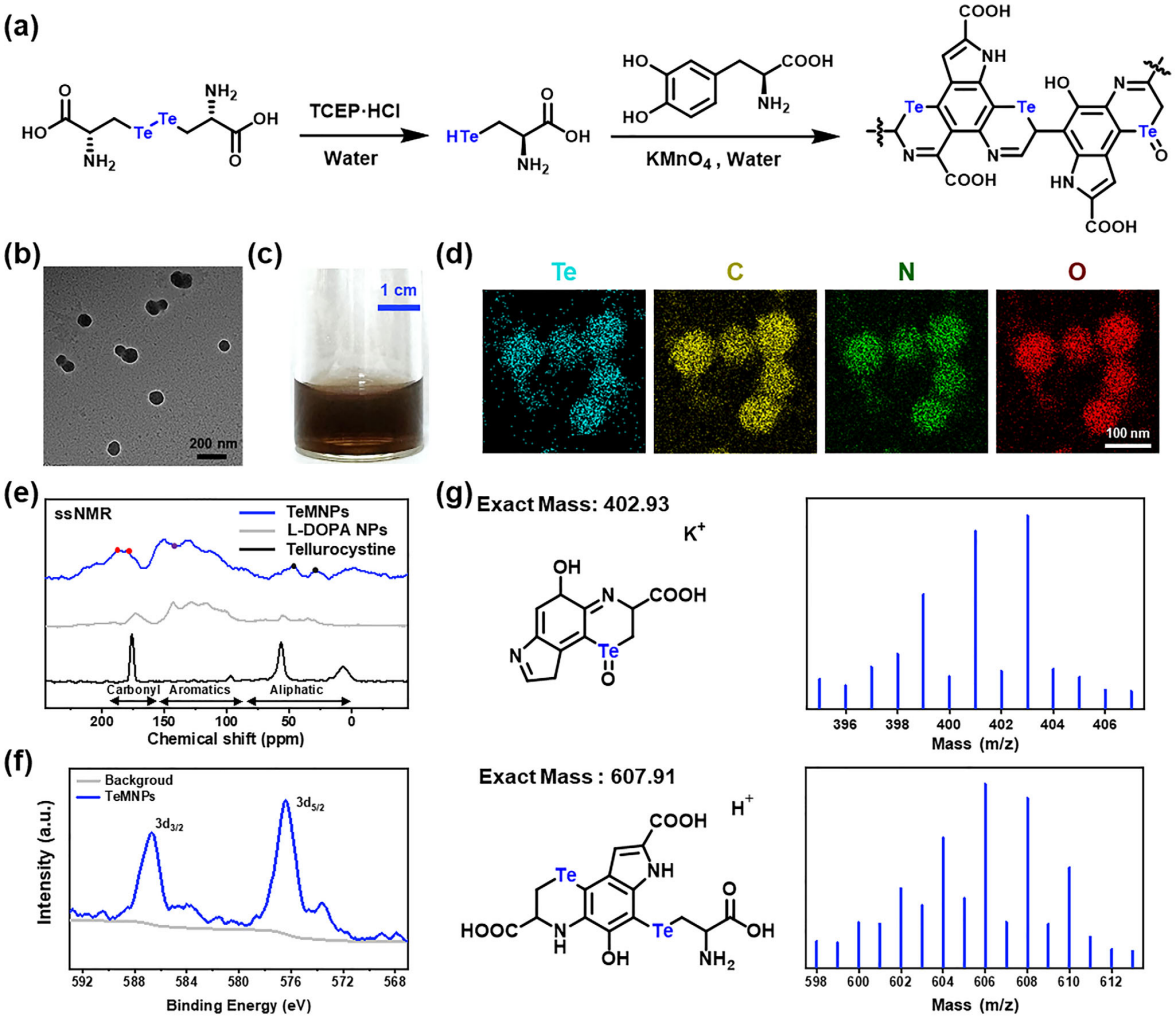
Synthesis and characterization of TeMNPs. (a) Synthetic route for TeMNPs via oxidative copolymerization of tellurocysteine and L‐DOPA in water at pH 7 (The chemical structure of TeMNPs was inferred from the detected reaction intermediates). (b) Representative TEM image of the synthetic TeMNPs. (c) Optical images of TeMNPs suspensions. (d) STEM‐HAADF image of TeMNPs and the corresponding EDX elemental mappings. (e) ^13^C ssNMR spectra of the synthetic TeMNPs from tellucysteine and L‐DOPA. (f) XPS spectra of the synthetic TeMNPs. (g) Mass spectra of intermediates during the synthesis of TeMNPs.

Inspired by natural biochemical processes in microorganisms, TeMNPs were strategically synthesized. Given the inherent instability of tellurocysteine (‐TeH) in atmospheric conditions compared to cysteine, L‐tellurocystine was initially prepared as a key synthetic intermediate. Tellurium powder was reduced with NaBH_4_ to generate Na_2_Te_2_, then reacted with β‐chloro‐L‐alanin, yielding L‐tellurocystine (Figure 1a) [19]. Nuclear Magnetic Resonance (NMR) spectroscopy, electrospray ionization mass spectrometry (ESI‐MS) data, and x‐ray photoelectron spectroscopy (XPS) verified the ditelluride structure of tellurocystine (Figures S1–S4). Notably, cysteine serves not only as the precursor for glutathione, a crucial intracellular antioxidant, but also polymerizes with dopaquinone under 5,6‐dihydroxyindole‐2‐carboxylic acid oxidase catalysis to form pheomelanin—an important biomacromolecule that helps cells combat oxidative stress damage, including radiation‐induced cellular damage [27, 28, 29, 30, 31, 32]. Inspired by this, we speculated that tellurocysteine may have similar biological activity, and its unique tellurium valence electron configuration may potentially endow the molecule with superior antioxidant properties and polymerization reactivity [23, 33]. Therefore, subsequent copolymerization with levodopa (L‐DOPA) produced the TeMNPs (Figure 1a). Homopolymerization of L‐DOPA alone produced control levodopa nanoparticles (L‐DOPA NPs), enabling comparative evaluation of the radioprotective properties imparted by Te incorporation.

Transmission electron microscopy (TEM) and dynamic light scattering (DLS) revealed that TeMNPs exhibited a uniform spherical morphology with a hydrodynamic diameter of ∼183 nm and a zeta potential of −24.6 mV (Figure 1b,c; Figure S5). Energy‐dispersive x‐ray spectroscopy (EDS) elemental mapping demonstrated homogeneous Te distribution throughout the spherical polymer architecture (Figure 1d). Inductively coupled plasma mass spectrometry (ICP‐MS) quantified the Te content in TeMNPs at ∼21 wt.% (Figure S6). The L‐DOPA NPs exhibited comparable particle size and zeta potential to TeMNPs (Figures S5 and S7), and broad‐band UV–vis absorption (Figure S8).

^13^C solid‐state nuclear magnetic resonance (ssNMR) was employed to verify the successful copolymerization of tellurocysteine and L‐DOPA. Three distinct spectral features changed in the ^1^ ^3^C ssNMR profile (Figure 1e): (a) the peaks at 188.2 and 179.0 ppm were assigned to the ─COOH groups of L‐DOPA and tellurocysteine, respectively, (b) a declining 143.1 ppm signal marking benzotellurazolyl *o*‐aminophenol generation, and (c) an expanding 55.0–29.0 ppm region reflecting aliphatic carbon enrichment [34]. X‐ray photoelectron spectroscopy (XPS) confirmed the presence of C─Te─C bonds within the polymer structure (Figure 1f) [35]. To directly confirm the covalent interaction between tellurocysteine and L‐DOPA leading to the formation of novel copolymer structures, we performed high‐performance liquid chromatography coupled with mass spectrometry (HPLC‐MS) analysis and characterization of the supernatant collected after 5 min of reaction (Figure 1g; Figure S9). The analysis of intermediates identified the telluroxide and benzotellurazine structure. Tellurium exists naturally as eight stable isotopes with mass numbers of 120, 122, 123, 124, 125, 126, 128, and 130 [36, 37]. Therefore, the mass spectrum displays a characteristic cluster pattern consisting of multiple fine isotopic peaks spanning over a dozen m/z values (Figure 1g; Figures S9 and S10). Previous studies have revealed that cysteine and selenocysteine primarily undergo mono‐substitution reactions with *o*‐benzoquinone (or catechol) structures [9, 27]. In contrast, tellurocysteine tends to participate in binucleophilic substitution reactions with these moieties. This can be attributed to the fact that tellurium is a soft nucleophile that preferentially interacts with soft substrates possessing conjugated structures through soft‐soft interactions [23, 38, 39]. This suggested that with identical numbers of reactive sites, the polymer structure could covalently incorporate more tellurium atoms, thereby enhancing the radiation energy attenuation capability of TeMNPs. Furthermore, the redox potential of catechol (∼ +570 mV) is likely higher than that of tellurocysteine in the neutral aqueous solution, resulting in competitive occupation of fewer reactive sites during the oxidative polymerization process [30, 40]. These provided additional evidence for the successful co‐polymerization.

### 2.2 The Radiation Attenuation, Tolerance, and Antioxidant Properties of TeMNPs

**FIGURE 2:**
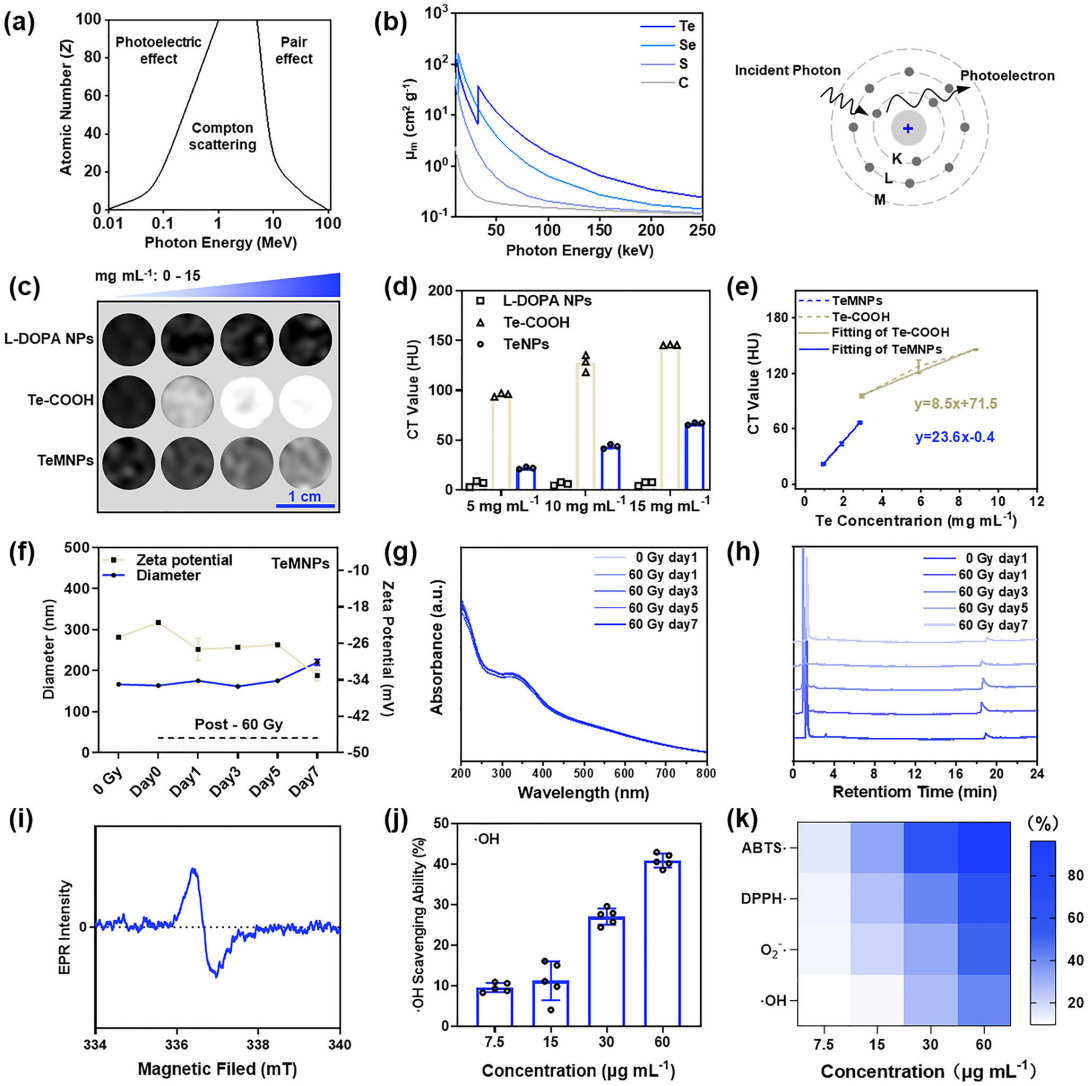
The radiation attenuation, radiation tolerance, and antioxidant capability of TeMNPs. (a) The relationship between three attenuation processes and atomic number and photon energy. (b) Mass attenuation coefficient (*µm*) of common elements including C, S, Se, and Te. The CT images (c) and CT values (d) of L‐DOPA NPs, TeMNPs, and tellurocystine were measured by computed tomography scanning at 80 kVp. (e) CT values of TeMNPs and tellurocystine with different Te concentrations. (f) The hydrodynamic size and zeta potential change of TeMNPs after 60 Gy irradiation within 7 days. (g) The UV–vis spectra of TeMNPs after 60 Gy irradiation within 7 days. (h) HPLC for irradiated TeMNPs to reveal potential alteration in the structure. (i) The EPR spectra of TeMNPs. (j) The ·OH scavenging ability of TeMNPs. (k) The scavenging ability of TeMNPs against various types of free radicals.

The primary interaction mechanisms for radiation photons in matter—namely, the photoelectric effect, Compton scattering, and pair effect—exhibit a positive correlation between their interaction cross‐sections and the *Z* of the target material (Figure 2a) [37]. These mechanisms cause attenuation of the incident beam energy through inelastic scattering and absorption, whereby energy is deposited into the medium [41]. The relationship between the photoelectric effect cross‐section 𝜎, *Z*, and photon energy *E* can be expressed as:

σ∝Z5/E3.5

For Compton scattering, the cross‐section 𝜎 and *Z* can be expressed as:

σ∝Z

In the photon (within 1.5 MeV)‐matter interaction regime, photoelectric effect and Compton scattering typically dominate. The Compton scattering cross‐section is linearly proportional to Z, while the photoelectric effect cross‐section scales with Z^4^^−^^5^. Thus, shifting the dominant interaction mechanism between a material and incident photons from Compton scattering to the photoelectric regime will lead to an exponential increase in the absorption of photon energy within the material. Compared to the elements carbon (C), sulfur (S), and selenium (Se), tellurium offers the most effective shielding against radiative energy (Figure 2b). The computed tomography (CT) value is a pivotal parameter for assessing a substance's capacity to shield against radiation. To this end, the potential shield ability of TeMNPs was measured using a medical CT scanner. As the concentration increased, the CT image brightness of TeMNPs gradually increased. The CT values (Hounsfield units, HU) of TeMNPs at three concentrations (5 mg mL‐1, 10, and 15 mg mL‐1) were 22.0, 43.6, and 66.7 HU, respectively, exceeding those of L‐DOPA NPs (Figure 2c,d). The values were also significantly higher than those of the selenium‐containing melanin (SeMNPs) we previously reported, which exhibited CT values of 18.5, 31.8, and 51.3 HU at concentrations of 5, 10, and 15 mg mL^−^^1^, respectively [41]. This suggested the incorporation of Te effectively enhanced the protective performance against radiation. The highest CT values were exhibited by tellurocystine (Te‐COOH), attributable to its elevated Te content (Figure 2d). However, the CT values exhibited a linear correlation with the Te mass concentration. The calculated x‐ray absorption coefficients versus Te mass concentration showed that TeMNPs (23.6 HU mg^−1^ mL) possessed higher x‐ray absorption coefficients than tellurium monomers (Tellurocystine, 8.5 HU mg^−1^ mL) (Figure 2e). The TeMNPs demonstrated a ∼ 3‐fold enhancement in energy absorption efficiency, which could result from multiple effects induced by polymeric structure [42]. Compared to tellurocystine, incorporating nitrogen‐doped conjugated heterocyclic moieties markedly improves molecular coplanarity [42]. Next, the extended π electron delocalization system facilitates more efficient photon energy absorption [43]. The trend of mass attenuation coefficients obtained from Monte Carlo simulations also indicates that upon incorporation of Te, the radiation attenuation capability of melanin is significantly enhanced (Figure S11), markedly surpassing that of the SeMNPs we previously reported [41].

The radiation energy absorption of materials gives rise to concerns regarding the material stability under high‐dose radiation. So, the stability of TeMNPs following irradiation with a high dose of 60 Gy γ‐ray was detected. A slight expansion of the particles was observed at 7 days, accompanied by a slight decrease in zeta potential (Figure 2f). This suggested the structure may have undergone minor alteration. To further investigate these minor alterations, various characterization methods continued analysis. The UV–vis spectra exhibited no alterations in absorption after the irradiation procedure during 7 days (Figure 2g; Figure S12). Proximally, the HPLC analysis of the supernatant irradiation treated‐TeMNPs also showed that the structure was not destructed (Figure 2h; Figure S13). The consistent Te content (Figure S14) preserved C─Te bonds in EDS mapping (Figure S15). Before irradiation, XPS analysis revealed that the TeMNPs contained tellurium in two distinct oxidation states: Te(IV), characterized by binding energies of 586.8 and 576.4 eV, and Te(II), with corresponding peaks at 584.2 and 573.8 eV [37]. Upon dispersion in water, irradiation with gamma rays leads to the radiolysis of water molecules, generating a range of reactive oxygen species (ROS), including hydroxyl radicals (·OH). These reactive species subsequently interact with the TeMNPs, triggering significant changes in their surface chemistry. Most notably, post‐irradiation XPS spectra revealed the near‐complete disappearance of the Te(II) characteristic peaks, indicating that the lower‐valent tellurium is preferentially involved in scavenging radiation‐induced ROS. (Figure S16). These collectively demonstrate TeMNP's exceptional radiation resistance, supporting their potential as radiation shielding materials.

Radiation‐induced ROS bursts in living cells, followed by cell death due to redox imbalance, is one of the major triggers of radiation‐related damages [34]. The semiquinone structure formed upon polymerization endowed TeMNPs with persistent free radicals, which have the ability to neutralize excess toxic free radicals within the cell (Figure 2i) [32, 44]. Therefore, TeMNPs may attenuate radiation‐induced cellular damage by supporting the intracellular antioxidant system. TeMNPs demonstrated a substantial capacity to scavenge ABTS and DPPH radicals, even at low concentrations 7.5 µg mL^−1^ (Figure S17). TeMNPs also exhibited remarkable scavenging effects for two common and harmful free radicals in cells: hydroxyl radicals (·OH) and superoxide anion radicals (O_2_^−^·) (Figure 2j,k; Figure S18). Consequently, the administration of TeMNPs emerges as a promising approach for enhancing biological resistance to radiation‐induced damage.

### 2.3 Radioprotective Effects Against γ‐Ray of TeMNPs in Living Cells

**FIGURE 3:**
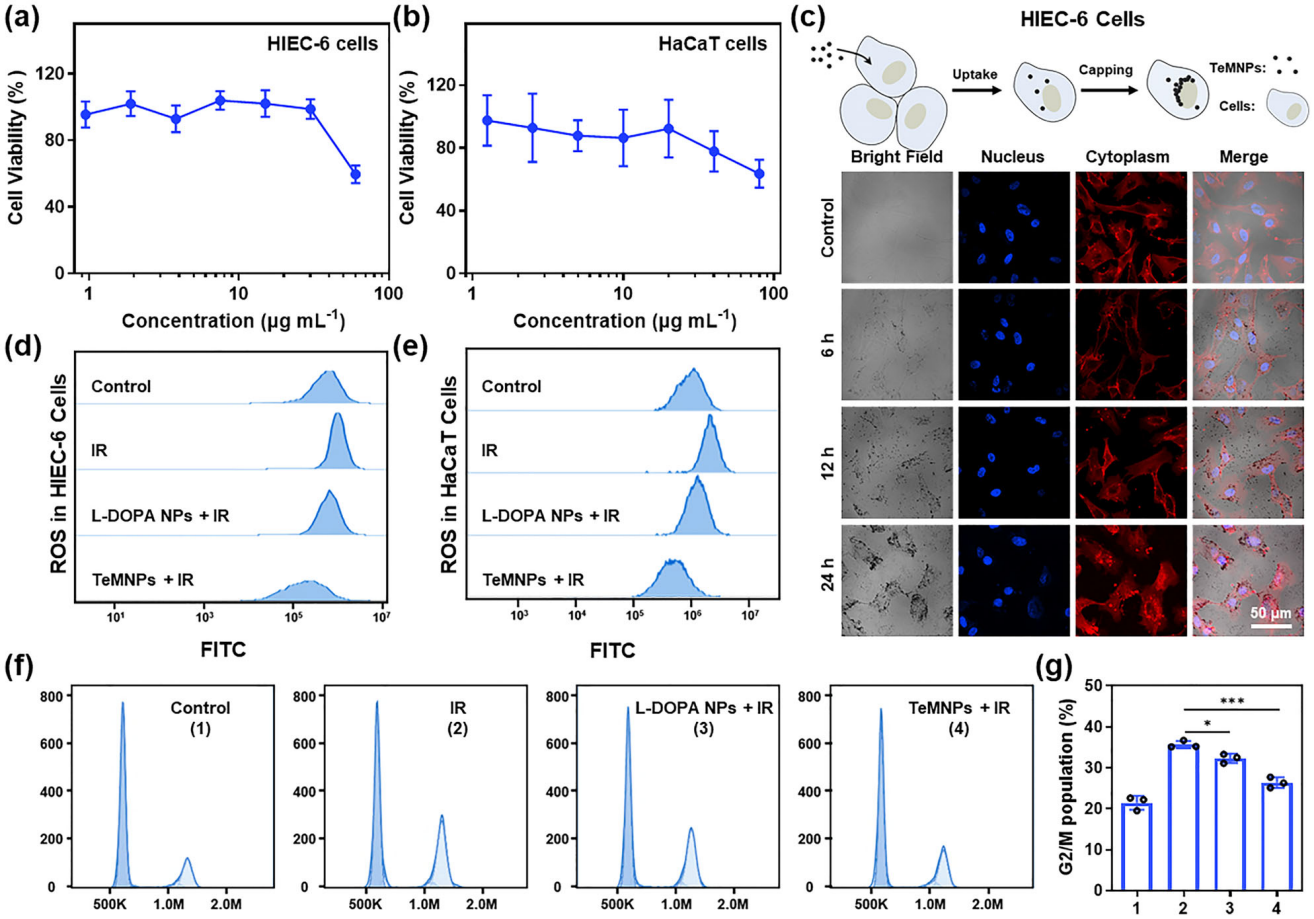
The radioprotection against γ‐ray of TeMNPs in HIEC‐6 and HaCaT cells. Cell viability of HIEC‐6 (a) and HaCaT (b) cells after 24 h treatment with TeMNPs at different concentrations. (c) The cellular uptake of TeMNPs in HIEC‐6 cells was detected by CLSM. ROS levels in HIEC‐6 (d) and HaCaT (e) cells after 24 h with different treatments were analyzed using flow cytometry. (f) Cell cycle distribution plots in HIEC‐6 cells with different treatments at 24 h. (g) The quantitative results of G2/M phase proportion. (^*^: p < 0.05, ^**^: p < 0.01, ^***^: p < 0.001).

To ascertain the potential of TeMNPs in mitigating the deleterious effects of radiation on living cells, we selected human intestinal epithelial cell‐6 (HIEC‐6) and human keratinocyte cells (HaCaT) for this study. These cell lines were chosen because the skin is the primary organ exposed to irradiation, and the intestines are among the most radio‐sensitive organs in the human body [45, 46, 47]. Following a 24 and 48 h treatment with TeMNPs, the 20 µg mL^−1^ was identified as the optimal subsequent treatment through a cell viability assay (Figure 3a b; Figure S19). Within a 24 h treatment, the cells exhibited continuous uptake of TeMNPs, eventually aggregating around the nucleus to form a perinuclear cap, mimicking the subcellular distribution of natural melanosomes (Figure 3c; Figures S20 and S21). Previous studies have indicated that the perinuclear cap structure formed by nanoparticles has the potential to mitigate the direct DNA damage caused by radiation through attenuation of the irradiation energy [9]. Thus, we believed the formation of perinuclear caps by TeMNPs with high CT values could offer a reliable refuge for DNA in the nucleus.

Radiation‐induced toxicity is initiated by intracellular ROS overgeneration and DNA damage caused by high‐energy deposition. Next, the fluorescent probe 2, 7‐dichlorodihydrofluorescein was employed to test the levels of ROS in the cells following various treatments. Subsequent analysis via flow cytometry indicated the ROS levels in the HIEC‐6 and HaCaT cells exposed to irradiation (IR) exhibited a marked increase compared to the control group (Figure 3d,e). The addition of TeMNPs proved effective in reducing the intracellular ROS levels after incubation with the cells (Figure 3d,e), suggesting that TeMNPs can inhibit oxidative stress and reduce cellular damage. Furthermore, it is well accepted that radiation‐induced DNA damage leads to the initiation of DNA repair, as evidenced by the arrest of the G2/M (Gap 2/Mitosis) phase in the cell cycle [34]. In this study, irradiation induced G2/M phase arrest in both cell lines, with the proportion of HIEC‐6 cells in G2/M phase increasing from 21% to 36% and that of HaCaT cells rising from 7% to 12% (Figure 3f,g; Figure S22). Further, the results demonstrated that the TeMNPs led to a significant decrease in the G2/M phase, with a reduction from 36% to 26% of HIEC‐6 cells (Figure 3f,g) and a reduction from 12% to 8% of HaCaT cells (Figure S22), respectively. This finding suggested that TeMNPs can effectively mitigate the cell cycle arrest induced by γ radiation. These outcomes were found to be superior to those observed with L‐DOPA NPs, a phenomenon that was attributed to the reduced DNA damage caused by γ‐rays that have undergone energy attenuation by TeMNPs.

### 2.4 In Vivo Distribution and Safety of TeMNPs via Oral Administration

**FIGURE 4:**
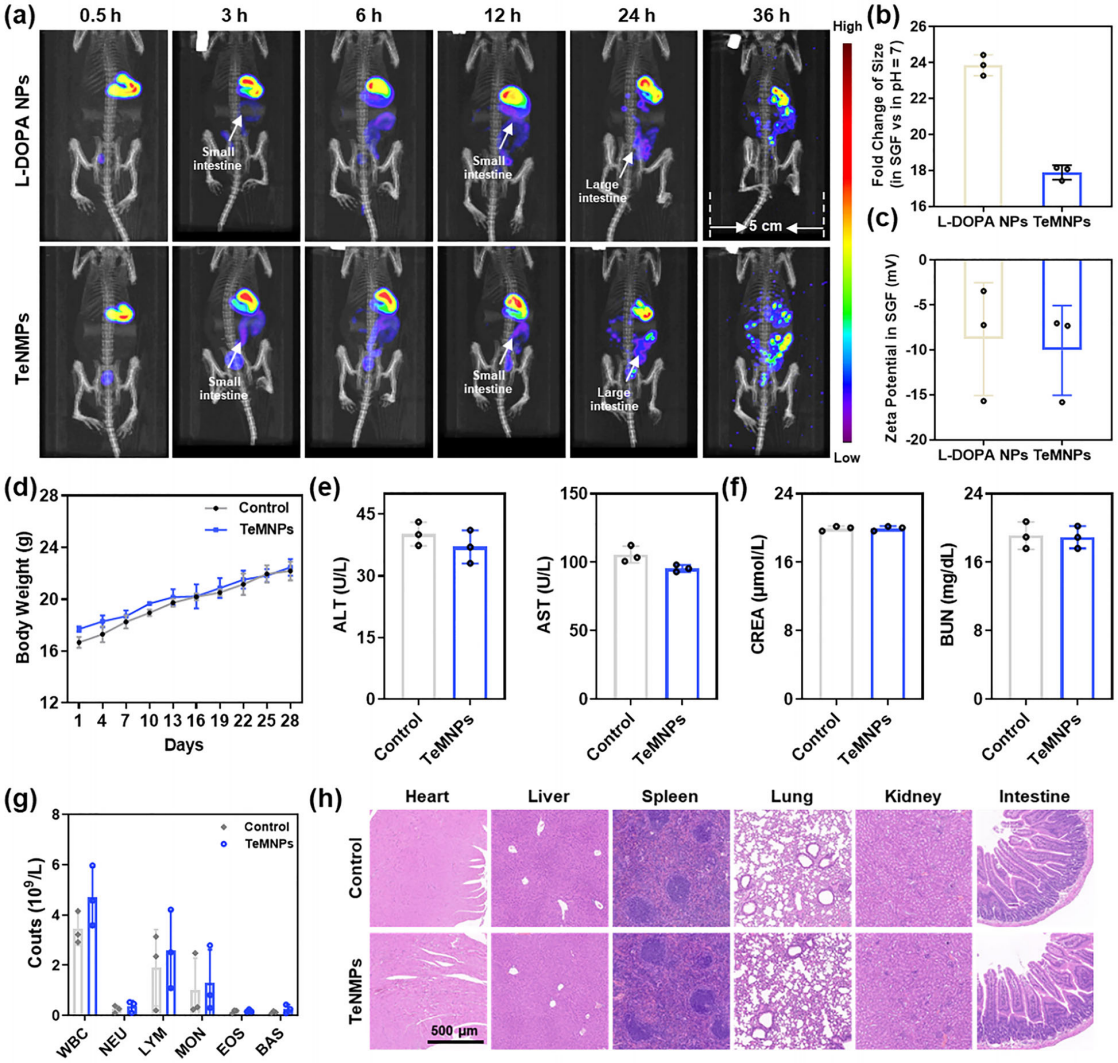
In vivo imaging and safety of TeMNPs. (a) SPECT‐CT images of the mice after oral administration of L‐DOPA NPs and TeMNPs at different time points. (b) The size change of L‐DOPA NPs and TeMNPs in simulated gastric fluid (SGF). (c) Zeta potential of L‐DOPA NPs and TeMNPS in SGF. (d) The mouse body weight change after oral administration of TeMNPs within 4 weeks. (e) Serum levels of hepatic function indices: ALT and AST. (f) Serum levels of renal function indices: CREA and BUN. (g) The counts of different types of white blood cells in the mice's blood. (h) Hematoxylin and eosin (H&E) staining of major organs, including liver, heart, spleen, lung, kidney, and intestine. n = 3 mice per group.

Inspired by the outstanding radioprotection capabilities of TeMNPs for intestinal cells in vitro, we further advanced their investigation at the animal level. The rapid intestinal excretion kinetics necessitate clinical application of sustained‐release dosage forms to prolong intestinal gastrointestinal lumen retention and absorption profiles [48]. In innovative intestinal nanotherapeutic platforms, gastrointestinal residence time serves as a key criterion for assessing drug design rationality [48, 49]. Therefore, using the clinically diagnostic radionuclide ^99m^Tc to label TeMNPs, we tracked their gastrointestinal retention time in mice using non‐invasive single‐photon emission computed tomography (SPECT‐CT). At 0.5 h post‐oral administration, TeMNPs were primarily distributed in the stomach (Figure 4a). The prolonged retention of TeMNPs and L‐DOPA NPs in the stomach may be due to their aggregation in the acidic environment (Figure 4b,c), which slows their transfer to the intestinal lumen. Remarkably, intense photon emissions were persistently detected in intestinal regions at 12–36 h post‐gavage, demonstrating prolonged gastrointestinal retention of TeMNPs (Figure 4a). This sustained presence likely results from the aggregated nanoparticles in the gastric environment, followed by subsequent dispersion and absorption in the intestinal tracts. Such extended residence time strongly indicated their potential as an orally deliverable intestinal radioprotectant.

Next, the biosafety of TeMNPs was evaluated. After oral administration of TeMNPs, the body weight of mice exhibited normal growth over 30 days (Figure 4d). Serum biochemical analysis revealed no significant changes in major liver function markers: alanine aminotransferase (ALT) and aspartate aminotransferase (AST) or kidney function markers: creatinine (CREA) and blood urea nitrogen (BUN) (Figure 4e,f) [50]. Additionally, there were no notable alterations in the number or function of white blood cells, red blood cells, or platelets in the mice (Figure 4g; Figures S23 and S24). No histopathological changes were observed in the major organs, including the heart, liver, spleen, lungs, kidneys, and intestines (Figure 4h), suggesting the good safety of TeMNPs.

### 2.5 Protective Effects on Radiation‐Induced Intestinal Injury in Mice

**FIGURE 5:**
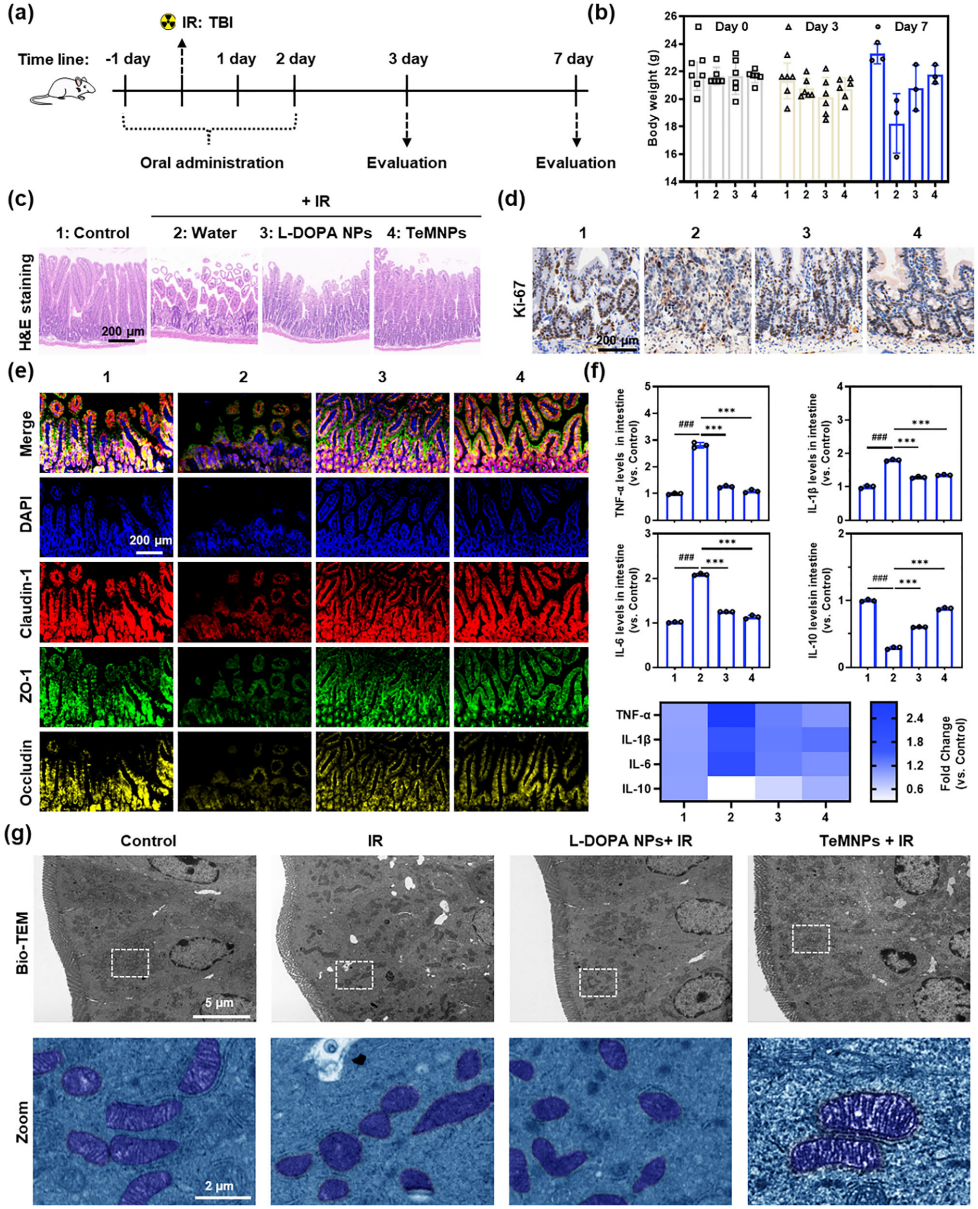
Therapeutic effects on radiation‐induced intestinal injury in mice. (a) Schematic illustration of therapeutic experiments with different groups in radiation‐induced intestinal injury models. (b) Mice's weight was measured after the indicated treatment. (c) Representative H&E staining images of intestinal tissue in each group at 7 days. (d) The proliferation‐related Ki‐67 was marked to label proliferative active cells. (e) Immunofluorescence images of claudin‐1, ZO‐1, and occludin in the intestinal tract. (f) Intestinal tissue inflammation levels measured by ELISA kits. (g) Bio‐TEM images of the mouse intestine with various treatments at 7 days. n = 6 mice per group. (^*^: *p* < 0.05, ^**^: *p* < 0.01, ^***^: *p* < 0.001).

To evaluate the protective effects of orally administered TeMNPs against radiation‐induced intestinal injury, mice subjected to total‐body irradiation were employed (Figure 5a). By 3 days post‐irradiation, the mice exhibited significant weight loss, which further progressed by 7 days. Oral administration of TeMNPs and L‐DOPA NPs partially mitigated this weight loss, with TeMNPs demonstrating a more pronounced protective effect (Figure 5b). Subsequently, intestinal tissues were collected for further analysis. In irradiation‐only mice, severe epithelial cell apoptosis and marked villus shortening were observed (Figure 5c; Figure S25). In contrast, TeMNPs treatment effectively counteracted radiation‐induced histopathological damage. Notably, the L‐DOPA NPs group began to show mild intestinal injury at 7 days (Figure 5c; Figure S25), indirectly highlighting the unique radioprotective mechanism of TeMNPs. Terminal deoxynucleotidyl transferase ‐mediated dUTP nick‐end labeling (TUNEL) assay further confirmed the ability of TeMNPs to suppress radiation‐induced DNA damage in intestinal cells (Figure S26).

The unique self‐renewal capacity of intestinal stem cells underpins the intestinal's remarkable ability to regenerate after injury [46]. Immunohistochemical staining for the cell proliferation marker Ki‐67 revealed that irradiation significantly impaired the regenerative potential of intestinal epithelial cells, ultimately leading to structural defects due to failed repair (Figure 5d). Notably, TeMNPs treatment effectively preserved intestinal stem cells' renewal capacity at levels comparable to healthy controls (Figure 5d). The intestinal barrier plays a pivotal role in maintaining homeostasis and preventing pathogen/toxin invasion. Tight junction proteins (claudin‐1, zona occludens 1 (ZO‐1), and occludin) are critical components of this barrier [41]. Immunofluorescence analysis demonstrated the marked down‐regulation of all three tight junction proteins post‐irradiation, indicating barrier disruption (Figure 5e). In contrast, the complete preservation of protein expression was observed in TeMNPs‐treated mice. Furthermore, treatment with TeMNPs could significantly suppress radiation‐induced pro‐inflammatory cytokine expression and enhance tissue anti‐inflammatory capacity (Figure 5f). These findings reinforced the reliability of their therapeutic potential.

Next, bio‐TEM was employed to investigate ultrastructural alterations in intestinal tissues. Compared with the control group, irradiation‐induced microvilli became loosely arranged without evident shedding in intestinal epithelial cells, and mitochondria exhibited marked pathological features, including significant size reduction, cristae disorganization (fusion or complete loss), and increased matrix density (Figure 5g). Notably, TeMNPs treatment preserved normal mitochondrial structural features, with clearly discernible cristae architecture and physiological matrix density (Figure 5h). These observations align with established findings that ferroptosis plays a pivotal role in radiation‐induced intestinal injury [51]. The mitochondrial abnormalities observed in irradiated groups represent morphological characteristics of ferroptosis [52, 53]. The characteristic features of ferroptosis primarily include the abnormal accumulation of intracellular free iron ions and excessive buildup of lipid peroxides [53]. TeMNPs may synergistically inhibit the ferroptosis process through multiple mechanisms. The catechol/quinone groups in their melanin‐like nanostructure can efficiently chelate free Fe(II), blocking the chain reaction of the Fenton process (Figure S27) [54, 55]. Meanwhile, the unique d‐orbital electrons of tellurium endow it with extraordinary coordination capabilities, enabling the formation of stable coordination complexes with iron to achieve precise regulation of intracellular iron metabolism [56, 57]. Glutathione Peroxidase 4 (GPx4) is the primary enzyme responsible for eliminating lipid peroxides within cells. With a redox potential of ‐850 mV (vs. Ag/AgCl), tellurocysteine possesses an intrinsically lower potential than selenocysteine (−640 mV vs. Ag/AgCl), the catalytic center of natural GPx4. This fundamental property is considered a key factor that contributes to the superior efficacy of the tellurium‐based GPx4 mimic (with tellurocysteine as its active center) in scavenging lipid peroxides [18, 33].

### 2.6 Transcriptomic Analysis of Radiation‐Induced Intestinal Injury in Mice After the Regulation of TeMNPs

**FIGURE 6:**
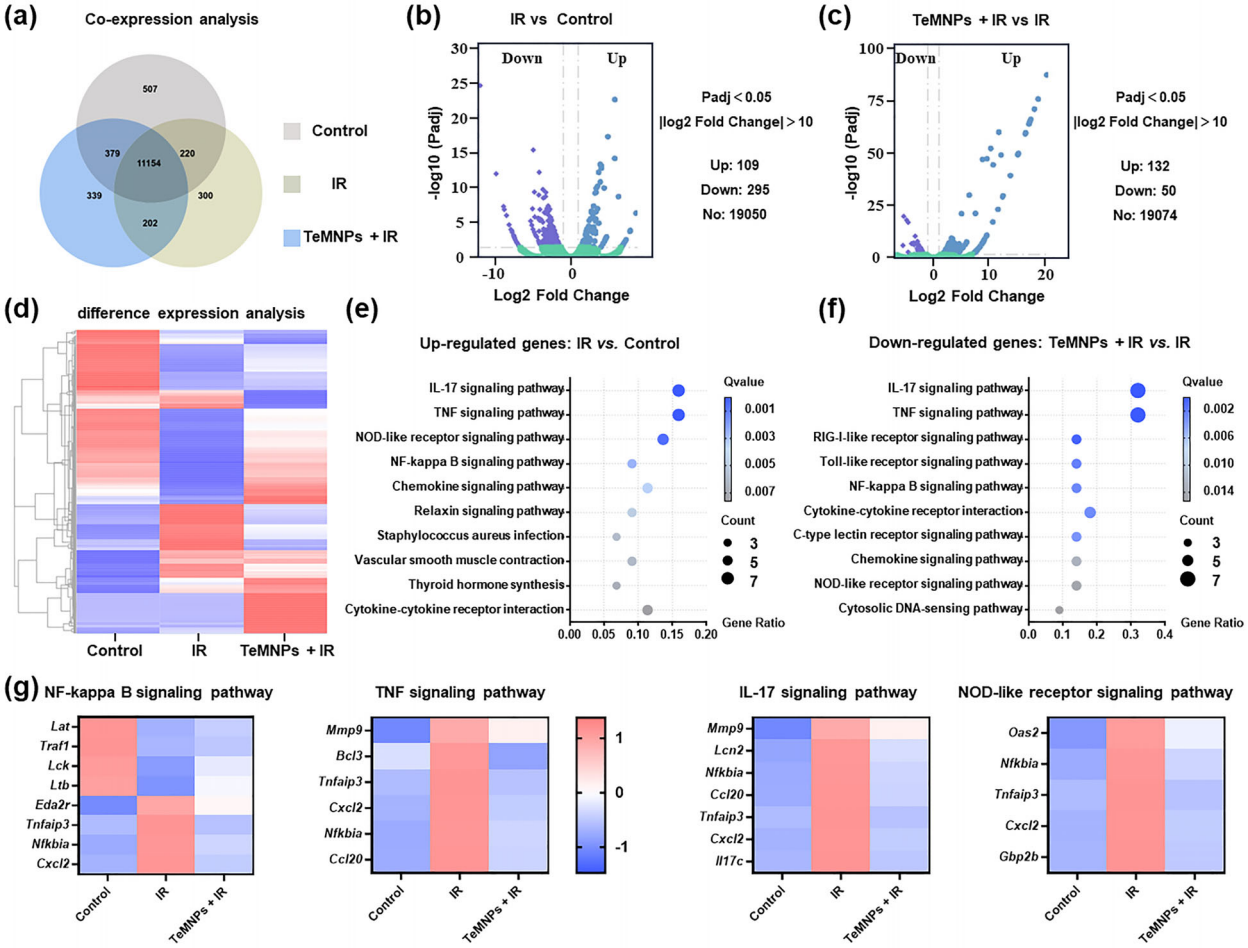
Analysis of the effect mechanism of TeMNPs at 7 days by RNA sequencing. (a) Venn diagram of whole‐transcriptome RNA‐seq analysis showing gene co‐expressed states among the control, IR, and TeMNPs + IR groups at 7 days. (b) Volcano plot of differentially expressed genes determined among the control, IR, and TeMNPs + IR groups. (d) Heatmap analysis of the differential gene expression in mice after being treated with TeMNPs. (e) KEGG pathways enrichment analysis of differential genes in the IR group versus the control group. (f) KEGG pathways enrichment analysis of differential genes in the TeMNPs + IR group versus the IR group. (g) Heat map of differentially expressed genes in NF‐κB signaling pathway, TNF signaling pathway, IL‐17 signaling pathway, and NOD‐like receptor signaling pathway.

To elucidate the potential radioprotective mechanisms of TeMNPs, we performed RNA sequencing analysis on murine intestinal tissues at 3 days and 7 days. Venn diagram analysis illustrated the overlapping gene expression profiles among the control group, irradiation group (IR group), and TeMNPs + IR group (Figure 6a; Figure S28). Volcano plot analysis demonstrated that, compared to the control group, there were significant differences in intestinal gene expression after different treatments at 3 days (Figure S29). Irradiation significantly upregulated 109 genes and downregulated 295 genes in intestinal tissues at 7 days (Figure 6b,c). Notably, oral administration of TeMNPs modulated this radiation‐induced dysregulation, resulting in upregulation of 132 genes and downregulation of 50 genes relative to the IR group (Figure 6b,c). The clustering analysis of differentially expressed genes further revealed that TeMNPs treatment exhibited a distinct pattern from the IR group, indicating the potential protective mechanisms of TeMNPs againt irrdiation (Figure 6d; Figure S30). Kyoto Encyclopedia of Genes and Genomes (KEGG) pathway enrichment analysis identified 4 inflammation‐associated signaling pathways: nuclear factor kappa‐B (NF‐Κb) signaling pathway, tumour necrosis factor (TNF) signaling pathway, interleukin 17 (IL‐17) signaling pathway, and nucleotide‐binding oligomerization domain‐like receptor (NOD‐like receptor) signaling pathway, that were significantly activated post‐irradiation (Figure 6e,f). TeMNPs treatment effectively suppressed the expression of these pro‐inflammatory pathways. Subsequent clustering analysis of the detailed pathway corroborated the inhibitory effect of TeMNPs on radiation‐induced intestinal inflammatory responses (Figure 6g).

## 3 Conclusion

In summary, we have successfully designed and synthesized a novel polymeric material through the innovative oxidative copolymerization of tellurocysteine. Due to the incorporation of tellurium (Te, *Z* = 52), the dominant interaction between natural melanin and incident photons shifts from Compton scattering to the photoelectric effect, significantly enhancing the radiation attenuation capability of TeMNPs. At a concentration of 5 mg/mL, the CT value of TeMNPs increases to 22.0 HU, much higher than L‐DOPA NPs and previous selenium‐containing melanin. Meanwhile, TeMNPs retain the antioxidant properties of melanin, exhibiting efficient scavenging activity against multiple types of free radicals. The unique polymeric architecture significantly improves radioprotective efficacy and structural stability under high‐dose radiation. Notably, TeMNPs demonstrate prolonged intestinal retention, effectively mitigating radiation‐induced intestinal damage, suppressing tissue inflammation, and preserving the integrity of the intestinal barrier. We believe this work establishes a novel paradigm for advancing amino acid‐based radioprotective materials through heavy‐element functionalization. In the future, TeMNPs will not only alleviate radiotherapy complications in clinical patients but may also provide support for space exploration.

## 4 Experimental Section

### 4.1 Reagents

β‐chloro‐L‐alanin was obtained from Shanghai Aladdin Biochemical Technology Co., Ltd. L‐3,4‐dihydroxyphenylalanine (L‐DOPA) was purchased from Shanghai Haohong Bio‐pharmaceutical Technology Co., Ltd. Potassium permanganate (KMnO_4_) was purchased from Beijing TongGuang Fine Chemicals Company. Stannous chloride (SnCl_2_) was purchased from Macklin. Na^99m^TcO_4_ was obtained from a ^99^Mo/^99m^Tc generator provided by Beijing Senke Pharmaceutical Co., Ltd.

### 4.2 Synthesis of TeMNPs and L‐DOPA NPs

TeMNPs: TeMNPs were synthesized at room temperature via oxidative copolymerization of L‐tellurocysteine and L‐DOPA using KMnO_4_ in ultrapure water (pH = 7). This templated polymerization reaction utilized pre‐formed L‐DOPA nanoparticles as seeds. Initially, L‐DOPA was dissolved in water and mixed with KMnO_4_ for 30 min. Subsequently, a pH‐adjusted (pH = 7) solution of tellurocysteine, prepared by reduction of tellurocystine, was introduced into the reaction system. After 24 h of continuous reaction, the products were collected by centrifugation (11 000 rpm for 10 min) and washed three times with HCl solution to exchange Mn^2+^ ions, followed by dialysis to remove free Mn^2+^ ions. Finally, the mass concentration of the nanoparticle solution was determined by lyophilizing a small aliquot overnight and weighing it using an analytical balance.

L‐DOPA NPs: L‐DOPA solution was mixed with 0.2 mol L^−1^ KMnO_4_ overnight. The product was purified by centrifugation and washed in HCl solution to exchange the Mn^2+^ ions.

### 4.3 Monte Carlo Simulation

Monte Carlo simulations were performed using the Geant4 software to evaluate the photon attenuation properties of TeMNPs thin‐sheet samples. The physics processes relevant to low‐energy photon interactions, including photoelectric absorption and Compton scattering. and Rayleigh scattering, were enabled using the standard electromagnetic physics list in Geant4. A monoenergetic unidirectional point photon source was employed, with photon energies ranging from 1 to 100 keV.

### 4.4 Cell Culture

The HIEC‐6 cells were purchased from Meisen CTCC (CTCC‐004‐0105, Hangzhou, China) (RRID: CVCL_6C21). The cells were cultured in DMEM/F12 medium (CTCC‐004‐0105‐CM, Hangzhou, China) supplemented with 10 ng mL^−1^ Epidermal Growth Factor, 100 U/mL penicillin, 100 µg/mL streptomycin (Gibco), and 10% fetal bovine serum (FBS; Biological Industries). The cells were incubated in a humidified incubator at 37°C with 5% CO_2_. HaCaT cells (RRID: CVCL_0038) were purchased from Wuhan Sunncell Biotechnology Co., Ltd, and cultured in Dulbecco's Modified Eagle Medium (DMEM) (VivaCell) containing 10% (v/v) FBS and 1% antibiotics (penicillin‐streptomycin) with high glucose, and maintained at 37°C with 5% CO_2_. Both cell types underwent Short Tandem Repeat (STR) profiling to confirm the absence of contamination.

### 4.5 Treatment for Radiation‐Induced Intestine Injury

All animal experiments were approved by the Institutional Animal Care and Use Committee (ethical number: BNUCC‐EAW‐20240403‐01; date of approval: 3‐31‐2024). Male BALB/c mice (6 weeks, body weight ∼20 g) were obtained from Beijing Vital River Laboratory Experimental Animal Technology Co. Ltd. The mice were housed in an air‐conditioned room maintained at 22 ± 1°C and 45 ± 5% humidity, with a 12 h light‐dark cycle. For intestine research, the mice were divided into the following four groups: (1) Control group, (2) IR group, (3) L‐DOPA NPs + IR group, and (4) TeMNPS + IR group. Mice were gavaged with 7.5 mg kg^−1^ of TeMNPs or L‐DOPA NPs twice within 48 h before irradiation. Afterward, mice were irradiated with 7.6 Gy. Next, mice continued to be gavaged with 7.5 mg kg^−1^ of TeMNPs or L‐DOPA NPs twice within 48 h after irradiation. Small intestines of mice were harvested 3 and 7 days after irradiation to assess prophylactic and therapeutic effects.

### 4.6 Statistical Analysis

All data are presented as the means ± standard deviation (S.D.) or means ± standard error of mean (S.E.M.). The statistical analysis of the data was determined by t‐tests (two‐tailed test) or one‐way analysis of variance (ANOVA) using SPSS Statistics 27. A p‐value of less than 0.05 was considered statistically significant.

## Funding

This project was supported by the National Key Research and Development Program of China (2022YFA1505900, 2023YFA0915300), the National Natural Science Foundation of China (22205026, 22471021, 52233012).

## Conflicts of Interest

The authors declare no conflicts of interest.

## Supporting information




**Supporting File**: advs75177‐sup‐0001‐SuppMat.docx

## Data Availability

The data that support the findings of this study are available from the corresponding author upon reasonable request.;
