# Supplementary material for: Biomimetic Polymerization of Tellurocysteine: Breaking the Natural Amino Acid Radioprotection Limitation
Source: Adv Sci (Weinh). 2026 Apr 7;13(37):e00010. doi: 10.1002/advs.202600010 (PMC13325932; doi:10.1002/advs.202600010)
Supplement: Supplementary file 1 — Supporting File: advs75177‐sup‐0001‐SuppMat.docx [file ADVS-13-e00010-s001.docx]

**Biomimetic Polymerization of Tellurocysteine: Breaking the Natural Amino Acid Radioprotection Limitation**

*Wei Chen^a, b^, Hanjie Zhu^a, c^, Yue Zhang^a, d^, Yuqing Qiao^a^, Ruotong Deng^a^, Huaping Xu*^, b^ and Wei Cao*^,a^*

[a] Key Laboratory of Radiopharmaceuticals of the Ministry of Education, College of Chemistry, Beijing Normal University, Beijing 100875, China
E-mail: [caowei@bnu.edu.cn](mailto:caowei@bnu.edu.cn)

[b] Key Lab of Organic Optoelectronics & Molecular Engineering Department of Chemistry, Tsinghua University, Beijing 100084, China
E-mail: [xuhuaping@mail.tsinghua.edu.cn](mailto:xuhuaping@mail.tsinghua.edu.cn)

[c] School of Physics and Astronomy, Beijing Normal University, Beijing 100875, China

[d] Institute of Catalysis for Energy and Environment, College of Chemistry & Chemical Engineering, Shenyang Normal University, Shenyang 110034, China

**Materials and methods**

**Characterizations**

Transmission electron microscopic (TEM) images were recorded using a FEI Tecnai Spirit 120 kV TEM at Tsinghua University. The zeta potential and dynamic light scattering (DLS) size of the nanoparticles in water was obtained on a Zetasizer Lab (Malvern Panalytical). Energy dispersive X-ray spectroscopy (EDS) mapping images were captured with a FEI Talos F200S. UV-Vis absorbance measurement was carried out on a Cary 60 UV-Vis Spectrophotometer (Agilent Technologies). Fluorescence spectra were collected on a Cary Eclipse Fluorescence Spectrophotometer (Agilent Technologies). X-ray photoelectron spectroscopy (XPS) spectra were analyzed by a Thermo Scientific K-Alpha. Electron paramagnetic resonance (EPR) spectra were measured on a Bruker EMXplus-6/1. Fourier transform infrared spectroscopy (FTIR) spectra were carried out on a Thermo Scientific Nicolet iS20 FTIR spectrophotometer. Inductively coupled plasma optical emission spectrometry (ICP-OES) was performed on an Agilent 5110 ICP-OES and a Thermo iCAP 7400 ICP-OES at Tsinghua University. Computed tomography (CT) images and Hounsfield Unit values were detected by a Philips IQon Spectral CT in Beijing Tongren Hospital. Confocal laser scanning microscopy (CLSM) images were acquired using a Nikon A1R microscopy system at Beijing Normal University and a Multi-SIM AXR multimodal super-resolution confocal microscope at Tsinghua University. Flow cytometry data was recorded via a BD Accuri^TM^ C6 Plus Flow cytometer. The fluorescence intensity of 96-well plates was monitored with the fluorescence microplate reader Tecan Infinite M200 PRO. Cells were irradiated by ^60^Co gamma irradiator (GM-11-03-A, Beijing Gamma high-tech Co., Ltd) in Beijing Normal University. TriFoil Triumph II microSPECT/CT equipment (Trifoil) was used for imaging studies. Radioactivity was determined on a PerkinElmer system (WIZARD^2^ 2480 Automatic γ-Counter).

**Synthesis of L-tellurocysteine**

First, 640 mg of tellurium powder and 380 mg of NaBH_4_ were added to 3 mL of ultrapure water in a sealed reaction vessel. The mixture was stirred at 50℃ for approximately 50 minutes, resulting in a deep purple Na_2_Te_2_ solution. After cooling to room temperature, 2 mL of thoroughly deoxygenated 3-chloro-L-alanine aqueous solution (pH=9) was added in multiple portions over 1 hour. The reaction was then stirred in darkness for 24 hours. Subsequently, the mixture was placed in an ice bath, and concentrated HCl was added dropwise to adjust the pH to 1, leading to the formation of black precipitates and H_2_Te gas. After warming to room temperature with continued stirring for 1 hour, the mixture was filtered through diatomaceous earth, and the solvent volume was reduced under vacuum. The solution pH was then adjusted to 6.5 using NaOH and maintained at 4℃ for 1 hour. The resulting precipitate was collected by filtration, washed repeatedly with water, and dried under vacuum to yield dark yellow tellurocystine as the final product.

**Stability detection after irradiation**

After exposure of TeMNPs to 60 Gy irradiation, a variety of characterization techniques were used to assess their stability after high-dose irradiation. In brief, after irradiation, the size, zeta potential, and absorption changes of TeMNPs were measured over 7 days using DLS and UV-Vis. The supernatant of irradiated TeMNPs was collected and subjected to UPLC analysis. The tellurium content in the supernatant was monitored by ICP-MS before and after irradiation.

**EPR test**

TeMNPs were separately loaded into quartz tubes, fixed in the resonator, and scanned using an EPR spectrometer (Bruker EMXplus-6/1). Nitroxide TEMPO (2,2,6,6-tetramethylpiperidine-1-oxyl) standards was used as reference materials. The measurement parameters were set as follows: Center Field, 3505.00 G; Power, 3.170 mW; Modulation Frequency (ModFreq), 100.00 kHz.

**CT test**

The centrifuge tubes (1.5 mL) were filled with TeMNPs, L-DOPA, and selenocystine at concentrations of 5, 10, and 15 mg/mL, respectively. CT images and Hounsfield Unit (HU) values were acquired using a Philips IQon spectral CT scanner at Beijing Tongren Hospital. Further analysis of the CT images was performed using PmsDView software. The imaging parameters were set as follows: 80 kVp, 13 mA.

**Measurement of intracellular ROS and cell cycle**

HIEC-6 cells and HaCaT cells were cultured in 12-well plates at a density of 1 × 10^5^ cells per well. After incubation for 12 h, the cells were pretreated with 100 µg mL^-1^ of TeMNPs and L-DOPA NPs solution for 24 h. Next, the cells were exposed to 6 Gy gamma ray (^60^Co radioactive source). Then, intracellular ROS levels were detected using the reactive oxygen fluorescent probe 2',7'-Dichlorodihydrofluorescein diacetate (DCFH-DA) (Beyotime Biotechonology Co. Ltd, Shanghai, China) and analyzed using flow cytometer.

For detection of cell cycle, the cells were unceasingly culture another 24 h after irradiation. Then, the cells were collected, fixed and stained according to the kit (Beyotime) manual provided by the reagent manufacturer, and analyzed by flow cytometry.

**Measurement of cellular uptake**

The uptake and intracellular fate of TeMNPs in HIEC-6 cells and HaCaT cells were observed and quantified using Multi-SIM AXR multimodal super-resolution confocal microscope (CLSM) and ICP-MS to assess the uptake. The cells were incubated with 10 μg/mL TeMNPs for 6 h, 12 h, and 24 h. For CLSM imaging, after cell fixation, nucleus and cytoplasm were stained with DAPI (Beijing Solarbio Science &Technology Co., Ltd.) and Dir (Beijing LABLEAD Inc.), respectively. For Te element concentration measurement, 1 × 10^6^ cells were collected and were quantitatively analyzed by ICP-MS.

**^99m^Tc Labeled NPs for SPECT Imaging**

100 µL of stannous chloride (SnCl_2_, 5 mg mL^-1^ in 0.1 M HCl) was added to a solution of TeMNPs and L-DOPA NPs (1 mL, 0.75 mg mL^-1^), which was then stirred with 3 mCi of Na^99m^TcO_4_ NaCl solution at room temperature for 30 min. The resulting ^99m^Tc-labelled TeMNPs and L-DOPA NPs solutions were purified by ultrafiltration several times to remove the free ^99m^Tc. Afterwards, the post-labelling radioactivity was then determined. For SPECT imaging, each mouse received 1.0 mCi (~ 15 mg/kg NPs) TeMNPs or L-DOPA NPs by gavage and was imaged with the microSPECT-CT system at different time points (30 min,180 min, 360 min, 12 h, 24 h and 36 h).

**Immunohistochemistry Assay and Immunofluorescence assay**

Small intestine slices (4 µm) were de-paraffinized and subjected to antigen retrieval using sodium citrate buffer or Tris-EDTA buffer. After blocking for 30 min at room temperature, endogenous peroxidase activity was reduced by incubation with 3% H_2_O_2_. The primary antibody of Ki-67 (K010075P, Solarbio), claudin-1 (GB15032, Servicebio), Zonula occludens-1 (GB151981, Servicebio), and occluding (GB111401, Servicebio) were then applied and incubated with the sections for 12 h at 4℃. The sections were incubated with the appropriate enzyme-labelled secondary antibody at room temperature. This was followed by incubation with DAB (3,3'-diaminobenzidine) and rinsed with PBST. Finally, the sllices were counterstained with haematoxylin.

**Elisa Assay**

In brief, 7 days after irradiation, mouse intestinal tissues were collected to determine the levels of inflammatory response-associated factors. The levels of TNF-α, IL-1β, IL-6 (Shanghai Enzyme-linked Biotechnology Co., Ltd) and IL-10 (SEKM-0010, Solarbio) were determined according to the protocol of the kit. Briefly, fresh samples were homogenized, the supernatant was collected after centrifugation, diluted and added to a 96-well plate, prior to the addition of enzyme-labelled antibodies. The plates were incubated at 37°C for 30 min and washed with PBS solution containing 0.05% Tween 20. After drying, biotin solution labelled with horseradish peroxidase was added and incubation was continued, then the plates were washed with PBS (containing 0.05% Tween 20) and reacted with tetramethylbenzidine (TMB). The reaction was terminated with dilute sulphuric acid solution and the absorbance at 450 nm was measured.

**RNA-seq analysis**

Mice in different treatment groups were anaesthetised and samples were collected 3 and 7 days after the mice were irradiated. The collected fresh small intestinal tissue washed 2 times with PBS and snap-frozen in liquid nitrogen for RNA‑sequencing.

RNA was extracted from the collected mouse intestinal tissues, and then Agilent 2100 bioanalyzer accurately detected the RNA integrity of the samples for strict quality control. Then the cDNA library was established by reverse transcription, and the effective concentration of the library was accurately quantified by qRT-PCR (the effective concentration of the library was higher than 1.5nM) to ensure the quality of the library. Finally, Illumina sequencing is carried out after pooling different libraries according to the requirements of effective concentration and target data volume.

**Safety evaluation of TeMNPs in vivo**

Mice were gavaged with 7.5 mg/kg of TeMNPs once daily for four days. Over the next 4 weeks, mice were tested for body weight. After 4 weeks, mice were euthanised to collect blood and major organs (heart, liver, spleen, lung, kidneys and small intestine) for safety assessment. Collected blood samples were used for serum biochemical, and blood cell and blood platelet analysis. Serum biochemical parameters of renal function, including creatinine (CREA), blood urine nitrogen (BUN), alanine aminotransferase (ALT), and aspartate aminotransferase (AST), were measured. The organs, including the heart, liver, spleen, lungs, kidneys, and intestine were collected, fixed in 4% paraformaldehyde, and stained with H&E for histopathological examination.

**Figure S1.** Synthetic route of tellurocystine.


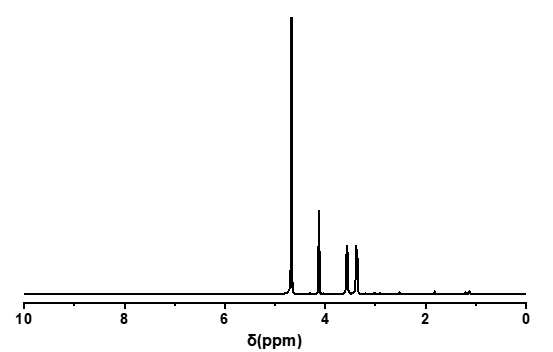


**Figure S2.** ^1^H NMR spectrum of tellurocystine.


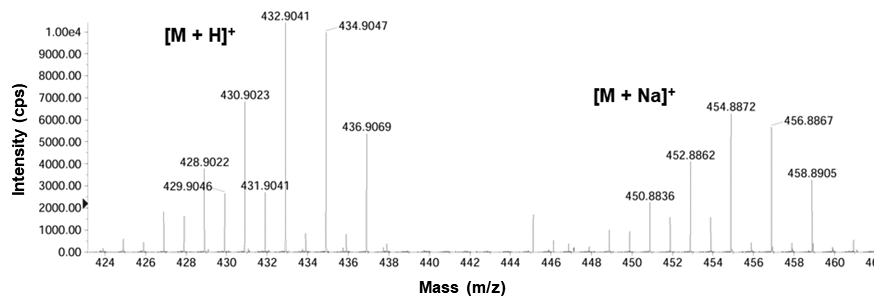


**Figure S3**. ESI-MS spectrum of tellurocystine.


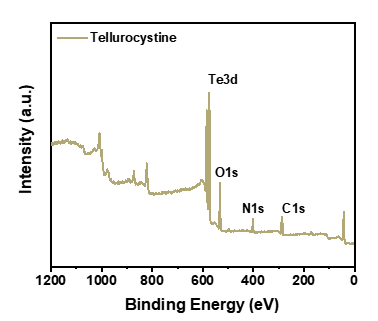


**Figure S4.** XPS spectra of the tellurocystine.


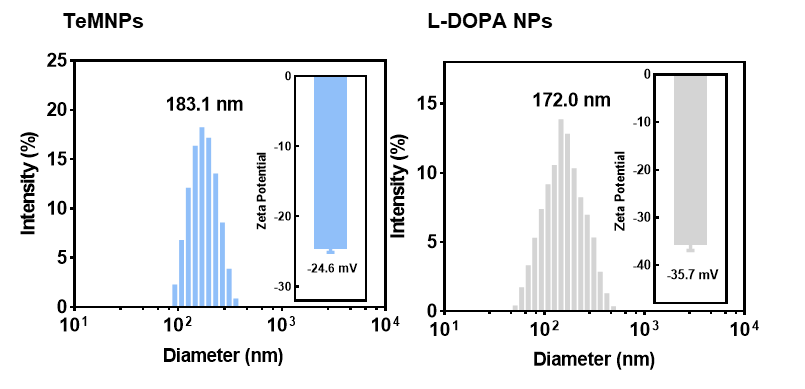


**Figure S5.** The hydrodynamic diameter and zeta potential of the TeMNPs and L-DOPA NPs.


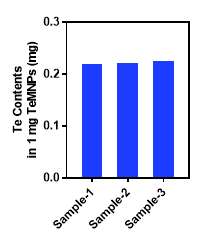


**Figure S6.** The Te contents in 1 mg TeMNPs.


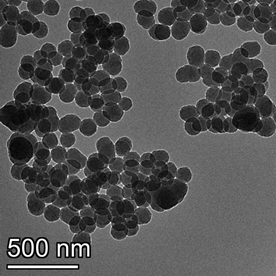


**Figure S7.** Representative TEM image of the L-DOPA NPs.


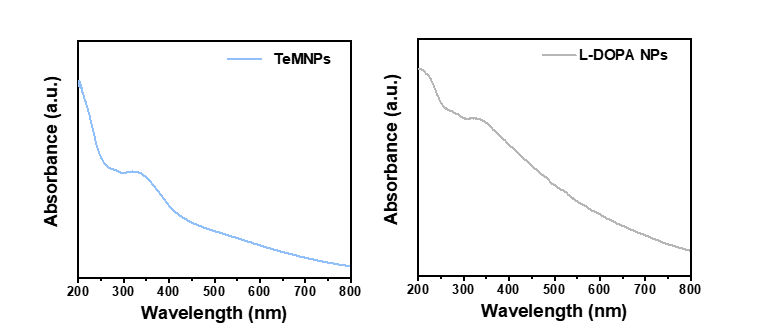


**Figure S8.** The UV-Vis spectra of TeMNPs and L-DOPA NPs.


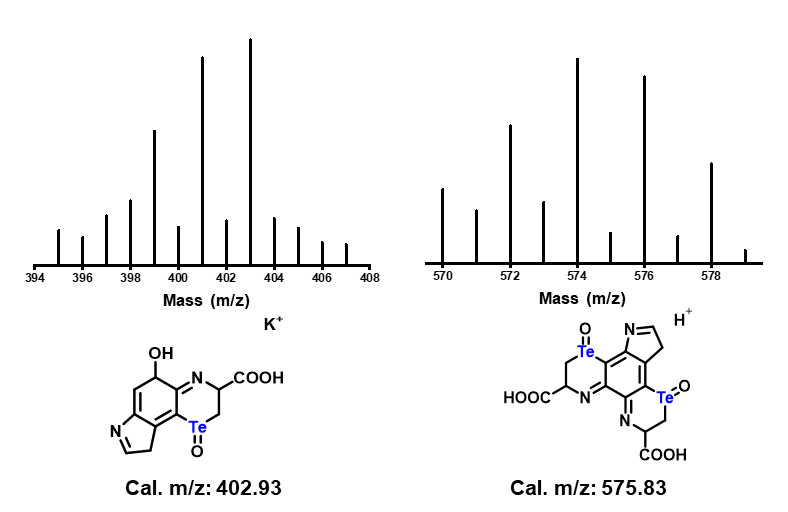


**Figure S9.** Mass spectra of intermediate products during the synthesis of TeMNPs.


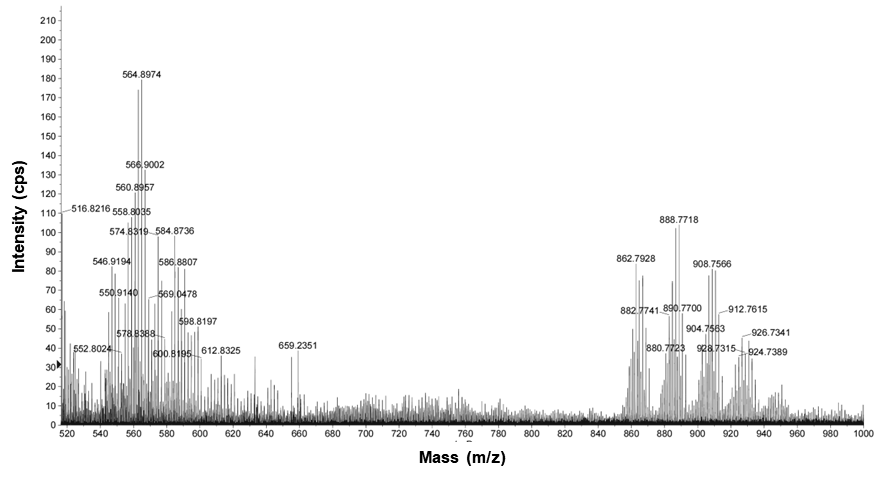


**Figure S10.** Mass spectra of intermediate products during the synthesis of TeMNPs.


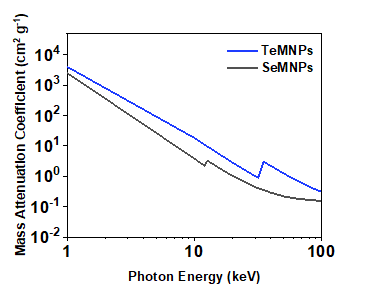


Figure S11. Mass attenuation curves of TeMNPs and SeMNPs from Monte Carlo simulation


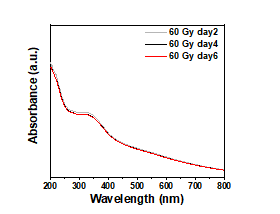


**Figure S12.** The UV-Vis spectra of TeMNPs after 60 Gy irradiation within 7 days.


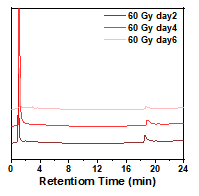


**Figure S13.** HPLC for irradiated TeMNPs to reveal potential alter in the structure.


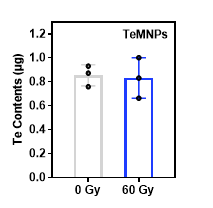


**Figure S14.** Te contents in liquid supernatant of TeMNPs with or without irradiation at 60 Gy.


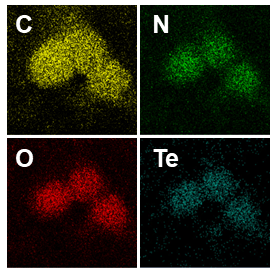


**Figure S15.** STEM-HAADF image of TeMNPs after 60 Gy irradiation.


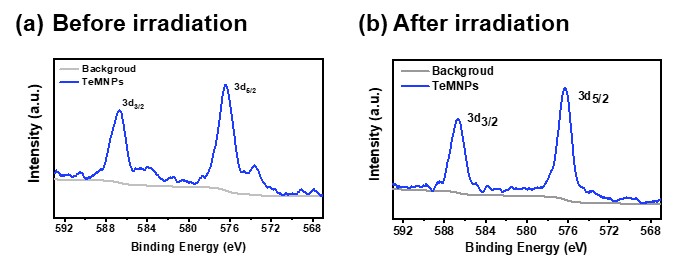


**Figure S16.** XPS spectra of TeMNPs with irradiation at 60 Gy.


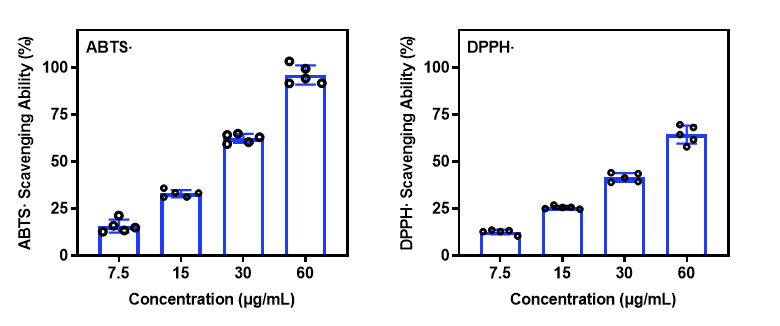


**Figure S17.** The ABTS· and DPPH· scavenging ability of TeMNPs.


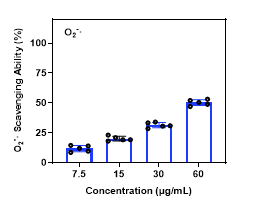


**Figure S18.** The O_2_^-^· scavenging ability of TeMNPs.


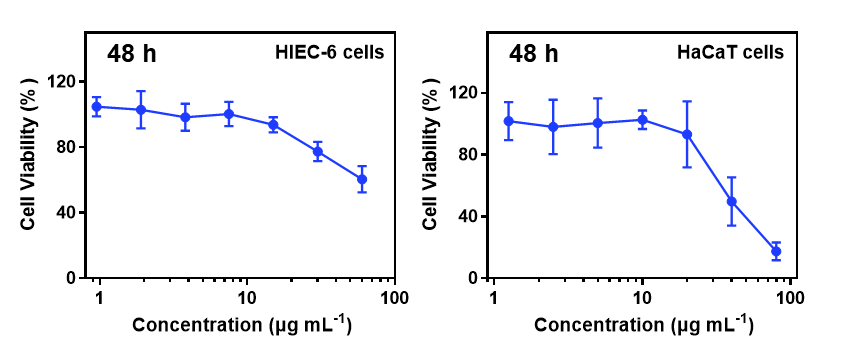


**Figure S19.** Cell viability of HIEC-6 and HaCaT cells after 48 h treatment with TeMNPs at different concentrations.


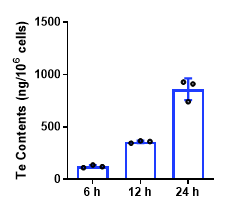


**Figure S20.** The Te contents in HIEC-6 cells detected by ICP-MS.


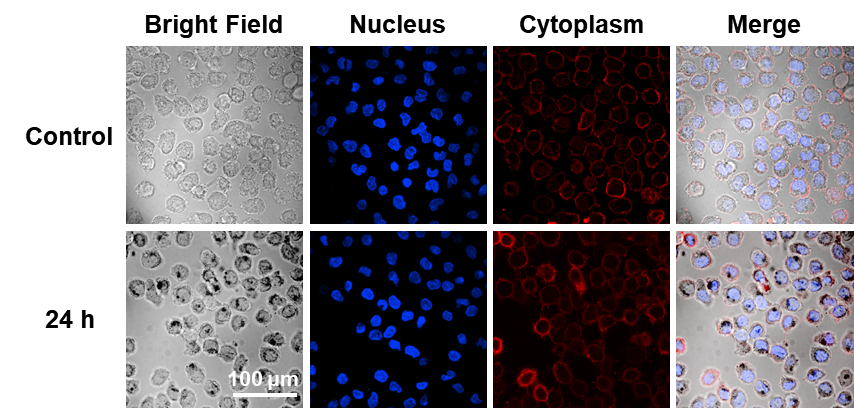


**Figure S21.** The cellular uptake of TeMNPs in HaCaT cells detected by CLSM.


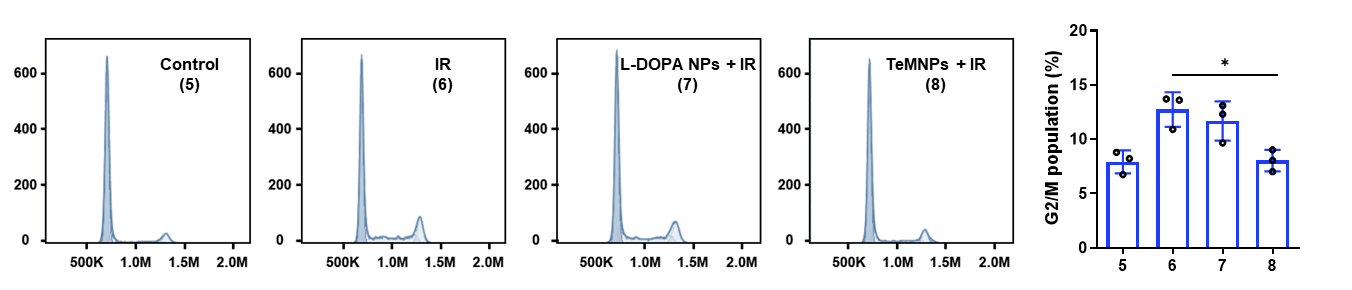


**Figure S22.** Cell cycle distribution plots and quantitative results in HaCaT cells with different treatment at 24 h.


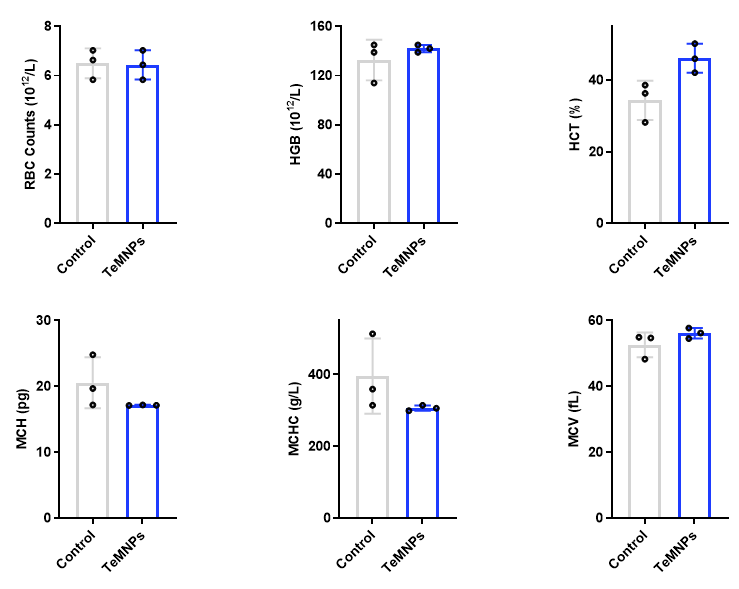


**Figure S23.** The change of red blood cell (RBC: red blood cells, HGB: hemoglobin, HCT: hematocrit, MCH: mean corpuscular hemoglobin, MCHC: mean corpuscular hemoglobin concentration. MCV: mean corpuscular volume).


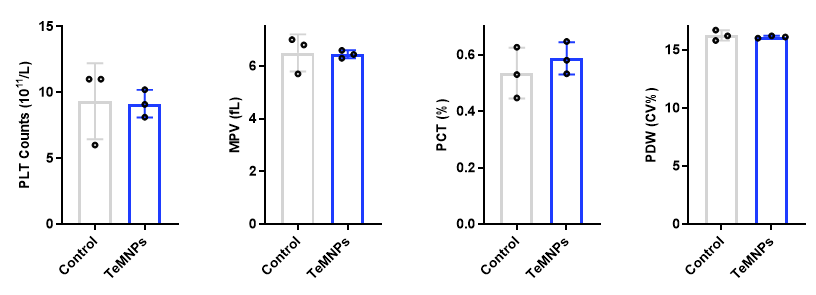


**Figure S24.** The change of blood platelet indices (PLT: blood platelet, MPV: mean platelet volume, PCT: plateletcrit, PDW: platelet distribution width).


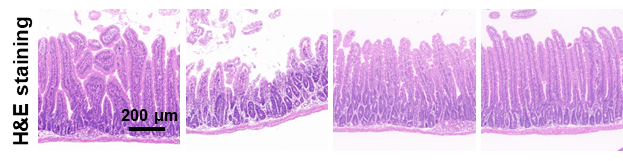


**Figure S25.** Representative H&E staining images of intestinal tissue in each group at 3 days.


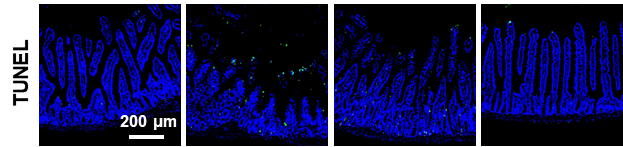


**Figure S26.** The DNA damage was stained by TUNEL and nuclei were stained with DAPI.

**Figure S27. Changes in the potential of TeMNPs after incubation with Fe(II).**


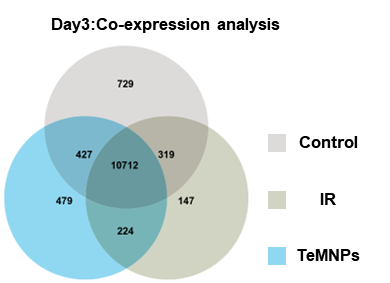


**Figure S28.** Venn diagram of whole-transcriptome RNA-seq analysis showing gene co-expressed states among the control, IR and TeMNPs + IR groups at 3 days.


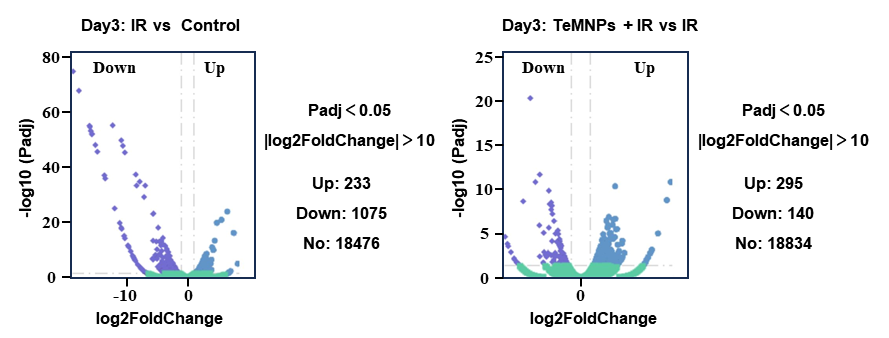


**Figure S29.** Volcano plot of differentially expressed genes determined among the control, IR and TeMNPs + IR groups at 3 days.


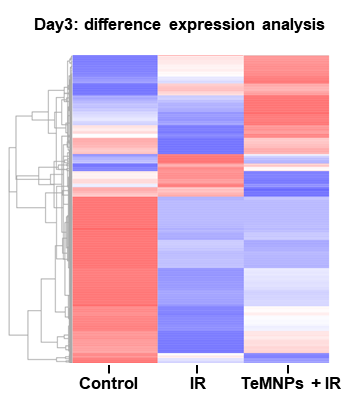


**Figure S30.** Heatmap analysis of the differential gene expression in mice after being treated with TeMNPs at 3 days.
